# Cerium Oxide Polishes Lithium Disilicate Glass Ceramic via a Chemical–Mechanical Process

**DOI:** 10.1055/s-0042-1753457

**Published:** 2022-09-05

**Authors:** Suparaksa Yamockul, Niyom Thamrongananskul

**Affiliations:** 1 Department of Prosthodontics, Faculty of Dentistry, Chulalongkorn University, Bangkok, Thailand

**Keywords:** cerium oxide, chemical–mechanical polishing, lithium disilicate, polishing paste

## Abstract

**Objective** The aim of the present study was to evaluate the chemical–mechanical polishing (CMP) effect of cerium oxide (ceria [CeO _2_ ]) as an abrasive to polish lithium disilicate glass ceramic.

**Materials and Methods** For the polishing experiment, 22 lithium disilicate glass ceramic samples were prepared, polished with sandpaper using a polishing machine, their surface roughness (Ra) was measured using a profilometer, and they were randomly divided into two groups ( *n* = 10). The samples were polished for 30 seconds with ceria paste with different ratios of deionized water:ceria by weight: 1:0.5 (C0.5) and 1:1 (C1) according to their group and the Ra values were determined. The Ra measurement was repeated after an additional 30 seconds of polishing until 120 seconds of polishing had been performed. The surface images of the postpolishing (120 seconds) samples were obtained using scanning electron microscopy (SEM) to evaluate the surface morphology. For the adsorption experiment, 10 lithium disilicate glass ceramic specimens were prepared and soaked in 50-mL deionized water. After 24 hours, the specimens were removed. Each liquid sample was divided in two halves. The first half was stored and ceria particles were added into the second half. After 24 hours, the solutions were filtered. The silicon concentration in the liquid samples was analyzed using inductively coupled plasma-optical emission spectrometry.

**Statistical Analysis** The difference in mean Ra value between groups was analyzed using two-way repeated analysis of variance (ANOVA) and the difference in mean silica concentrations before and after adding ceria particles was analyzed using the paired *t* -test. A *p* -value of <0.05 was considered statistically significant.

**Results** Ra decreased as the ratio of ceria and polishing time increased. The surface morphology of the samples analyzed by SEM corresponded with their Ra values. The mean silicon concentration after adding ceria particles was significantly decreased ( *p* < 0.05).

**Conclusion** Using a ceria-polishing paste to polish lithium disilicate glass ceramic generates a significantly smoother surface compared with baseline roughness. Moreover, CeO _2_ has a mechanical action and chemical reaction with lithium disilicate glass ceramic. Therefore, it can be used as a CMP paste to create a smooth surface.

## Introduction

Polishing is a process of wear on the surface of one material by another material to produce a smooth surface. 1 Polishing is required to restore a smooth surface after the final adjustment of dental restorations. An inadequately polished surface leads to gingival inflammation, increased dental plaque accumulation, wear of the opposing and adjacent teeth, and reduces the restoration strength and esthetics. 2 3 4 5 6 7 The current polishing modalities used in dentistry are mechanically based. However, there is a polishing process that combines a mechanical process and chemical reaction together called chemical–mechanical polishing (CMP).

CMP removes material using chemical and abrasive action complement each other to achieve a highly smooth surface. It is different from the purely mechanical process and purely chemical removal process. The aim of CMP is to minimize or eliminate direct material removal by either mechanical abrasion or by chemical etching. Mechanical removal, such as scratching, can cause severe damage to the surface. Corrosion occurs with chemical etching. The abrasives used in CMP must chemically react with the polished surface. 8

The CMP model originated from glass polishing in which cerium oxide (ceria [CeO _2_ ]) was used as an abrasive. It is considered the most effective abrasive for polishing glass. 9 The CMP occurring between CeO _2_ and silica (SiO _2_ ) have been investigated. Previous studies reported that silica on the glass surface and ceria abrasive particles can form surface functional groups that temporarily attach to each other. 10 11 12 These studies demonstrated that the formed layer is removed by the abrasive plowing of the slurry particles, exposing a new unreacted surface. Polishing is thought to occur, as the ceria particles repeatedly remove the silica network at the molecular scale.

All-ceramic restorations are widely used in dentistry because of their esthetic appearance. One of these materials is lithium disilicate glass ceramic (Li _2_ Si _2_ O _5_ or 2SiO _2_ –Li _2_ O) that contains 57 to 80% silica as the main component. 13 14 Because the main component of glass and lithium disilicate glass ceramic are silica, we hypothesized that CeO _2_ would react with the silica in lithium disilicate glass ceramic, and thus could be used as a CMP agent. The aims of the present study were to evaluate the CMP effect of CeO _2_ as an abrasive to polish lithium disilicate glass ceramic, and determine if silica would adsorb to CeO _2_ . The null hypotheses were that there would be no significant difference in the surface roughness (Ra) of lithium disilicate before and after polishing with ceria polishing paste and there would be no significant difference in silica concentration before and after adding ceria particles in solution.

## Materials and Methods

### Polishing Experiment

Twenty-two samples were used in this study. The sample size calculation was performed using the data from our pilot study. Lithium disilicate samples (7-mm long, 6-mm wide, and 5-mm thick) were prepared by cutting the blocks (HT A1, IPS e.max CAD, Ivoclar Vivadent, Schaan, Liechtenstein) using a low-speed precision cutting machine (IsoMet, 1000 No. 11–2180, Buehler, Illinois, United States), ultrasonically cleaned with deionized water for 5 minutes (CP360 Powersonic, Crest Ultrasonics, New Jersey, United States), rinsed with deionized water, and dried and fired in a furnace (Programat P300, Ivoclar Vivadent, Schaan, Liechtenstein) per the manufacturer's directions. After firing, the samples were cooled in the furnace. Each sample was embedded in a polyvinyl chloride pipe with epoxy resin. The position of the sample was set at the center on the pipe's surface. After the epoxy resin had completely hardened, a registration mark was made at the bottom of the pipe (4-mm wide and 6-mm deep) to allow the specimen to be aligned at the same position during multiple roughness measurements.

The specimens (six specimens/round) were polished for 5 minutes with 180 grit silicon carbide sandpaper (3M Wetordry abrasive sheet, 3M, Minnesota, United States) by a polishing machine with an automatic head (NANO 2000 grinder-polisher with FEMTO-1000 polishing head, Pace Technologies, Arizona, United States). During polishing, the samples and sandpaper were rotated at 200 rpm in the opposite directions. The pressure applied on the samples was set at 1 kg/cm ^2^ . New sandpaper was used each round. The polished specimens were ultrasonically cleaned in deionized water for 5 minutes, rinsed with deionized water and dried.

The Ra of the specimen was measured using a profilometer (Talyscan 150, Taylor Hobson, Leicester, United Kingdom) to determine the baseline roughness. Five 2-mm measurements were taken at the center of the sample (cut-off value of 0.25 mm and stylus speed of 0.5 mm/s). The vertical distance between each transverse measurement was 0.4 mm. The sample was then rotated 90 degrees and remeasured using the same procedure. The Ra values were averaged to generate a mean Ra value per sample.

After the baseline roughness evaluation, the samples were randomly divided into the C0.5 and C1 polishing paste groups ( *n* = 10).

### Polishing Paste Preparation

Deionized water was used as a lubricant. Ceria (CeO _2_ powder, <5 µm diameter particles, 99% trace metals basis, Sigma-Aldrich, Merck KGaA, Darmstadt, Germany) was used as the abrasive in the present study. The polishing pastes were prepared using different ratios of deionized water:ceria by weight: 1:0.5 (C0.5) and 1:1 (C1). The polishing pastes were prepared by weighing the components to within 0.0001 g using an analytical balance (GR 200, A&D, Tokyo, Japan) based on each group's composition and mixed using a spatula for 5 minutes. The mixtures were loaded into a syringe (0.1–1 mL scale; Slip-tip disposble tuberculin syringe, Medline Industries, Illinois, United States). The polishing pastes were used within 12 hours.

### Polishing Method

The C0.5 and C1 group samples were polished with their respective pastes. Each polishing paste (0.05 mL) was injected on the center of the specimen and then polished using a felt wheel (Felt wheel, Jota, Ruthi, Switzerland, 2.2-mm diameter) on a micromotor (Micromotor and handpiece, Saeyang microtech, Daegu, Korea) for 30 seconds, ultrasonically cleaned in deionized water for 5 minutes, rinsed with deionized water and dried. A new felt wheel was used for each group. The micromotor speed was set at 6,000 rpm, calibrated using a tachometer (Digital tachometer, RS components Ltd., Corby, United Kingdom). The polishing force was 0.39 N (equal to 40 g hand force). The operator was calibrated using a precision scale before and during the procedure. The calibration was repeated for every 10 specimens. 15 All polishing procedures were performed by one operator. After polishing, the specimens were ultrasonically cleaned in deionized water for 5 minutes, rinsed with deionized water and dried.

After polishing, the Ra of the specimen was measured using a profilometer as described for the baseline roughness measurement. After the measurement, the samples were ultrasonically cleaned in deionized water for 5 minutes, rinsed with deionized water and dried.

The Ra measurement was repeated after an additional 30 seconds of polishing until 120 seconds, that is, measured after 30, 60, 90, and 120 seconds of polishing, the polishing had been performed.

### Surface Roughness Measurement

The Ra of the polished surface was measured using a profilometer using the same procedure at the same position as at the baseline roughness measurement.

### Scanning Electron Microscopy Analysis

Two samples from each postpolishing (120 seconds) group and two unpolished samples with the baseline roughness were removed from the epoxy resin and ultrasonically cleaned in deionized water for 5 minutes, rinsed with deionized water, and dried. The specimens were mounted on adhesive coated aluminum stubs (1 sample/stub) and gold sputter-coated (100 second, 50 mA) using a sputtering device (JFC-1200 Fine Coater, JEOL, Tokyo, Japan). The surface images were taken using an electron microscope (Quanta 250 FEG scanning electron microscope, FEI, Oregon, United States) with 20 kV accelerating voltage and 500X magnification to evaluate the surface morphology.

### Statistical Analysis

The data were statistically analyzed using two-way repeated analysis of variance (ANOVA) followed by Bonferroni correction to compare the differences in mean Ra values between groups (SPSS version 26.0 for Windows, SPSS, Chicago, Illinois, United States). A *p* -value of <0.05 was considered statistically significant.

### Adsorption Experiment

#### Instrument Preparation

To avoid the dissolution of silicate ions, no glassware was used in this study. Nitric acid (10% by volume) was prepared from diluted nitric acid (70% by volume) (Ajax Finechem, Thermo Fisher Scientific Inc., Massachusetts, United States) with deionized water. The instruments used in the study were soaked in the acid solution for 24 hours to remove the residual ions on the instruments' surface, rinsed with deionized water thrice and dried in a hot air oven (60°C) for 4 hours.

#### Specimen Preparation

Ten samples were used in this study. The sample size calculation was performed using data from our pilot study. Lithium disilicate blocks (HT A1, IPS e.max CAD, Ivoclar Vivadent) were cut transversely into 2-mm thick specimens using a low-speed precision cutting machine (IsoMet, 1000 No. 11–2180, Buehler). The blade rotation speed was 300 rpm. The specimens were polished with 600 grit silicon carbide sandpaper (3M Wetordry abrasive sheet, 3M) by a polishing machine (NANO 2000 grinder-polisher, Pace Technologies, Arizona, United States) for 2 minutes. During polishing, the sandpaper was rotated at 200 rpm. The polished specimens were ultrasonically cleaned in deionized water for 5 minutes (CP360 Powersonic, Crest Ultrasonics), rinsed with deionized water and dried. The specimens were fired in a furnace (Programat P300, Ivoclar Vivadent) as per the manufacturer's directions. After firing, the specimens were cooled in the furnace.

#### Adsorption Analysis

The specimens were soaked in 50-mL deionized water (1 specimen/container, *n* = 10). The plastic containers were covered with a cap, sealed with parafilm (Parafilm M laboratory wrapping film, Bemis Company, Inc., Wisconsin, United States) and placed on a magnetic stirrer (Yellow MAG HS7, IKA, North Carolina, United States) for 24 hours at room temperature (25°C).

After 24 hours, the specimens were removed. Each liquid sample was divided into two halves by drawing 25 mL of the solution and transferred into a new container using a syringe (50-mL syringe, NIPRO, Osaka, Japan). The first half was stored in a closed container, capped and sealed with parafilm and placed in a refrigerator (4°C). Ceria particles (5 g, CeO _2_ powder, <5-µm diameter particles, 99% trace metals basis, Sigma-Aldrich, Merck KGaA) were added into the second half of each sample. The containers were capped, sealed with parafilm and left to equilibrate on a magnetic stirrer for 24 hours at room temperature (25°C).

After 24 hours, the solutions were filtered through a 0.22-μm membrane filter (tube top vacuum filter system, 0.22-µm pore, 50 mL, Corning, New York, United States), closed with a cap, sealed with parafilm and placed in the refrigerator (4°C).

#### Inductively Coupled Plasma-Optical Emission Spectrometry

In this assay, the blank test was deionized water. The concentration of silicon (Si) in the liquid samples was analyzed using an inductively coupled plasma-optical emission spectrometry analyzer (ICP-OES Optima 7300 DV, PerkinElmer, Inc., Massachusetts, United States). The analysis was repeated thrice per sample. The silicon concentrations were averaged to generate the mean value per sample.

### Statistical Analysis

The data were statistically analyzed using the paired *t* -test to compare the difference in mean silicon concentrations before and after adding ceria particles (SPSS version 26.0 for Windows, SPSS). A *p* -value of <0.05 was considered statistically significant.

## Results

**Fig. 1:**
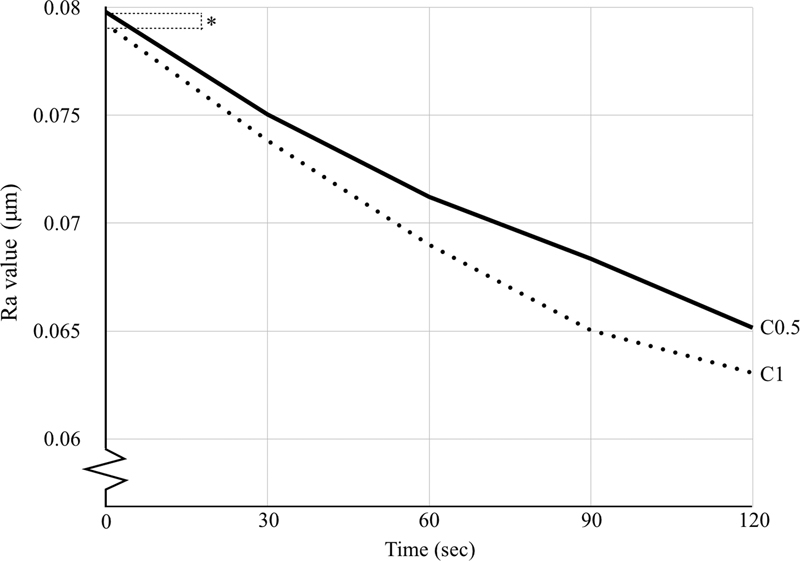
The mean Ra value of the groups before and after polishing for 30, 60, 90 and 120 seconds. Asterisks (*) represent no significant difference between groups ( *p* > 0.05) by two-way repeated ANOVA and Bonferroni correction. ANOVA, analysis of variance; Ra, surface roughness.

The results of the polishing experiment comprising the mean Ra values, standard deviations, and significant differences between the groups are presented in Table 1 . The mean Ra values were not significantly different between the groups at baseline ( *p* > 0.05). Within each group, the mean Ra values significantly decreased as the polishing time increased ( *p* < 0.05; Fig. 1 ). Comparing the groups, the C1 group demonstrated a significantly lower mean Ra value than the C0.5 group ( *p* < 0.05).

**Table 1:** Mean Ra values of the groups at baseline and after polishing for 30, 60, 90, and 120 seconds

Group	Ra value (μm) and standard deviation				
	Baseline	30 seconds	60 seconds	90 seconds	120 seconds
C0.5	0.07979 ± 0.00197 ^A^	0.07504 ± 0.00060	0.07122 ± 0.00073	0.06835 ± 0.00072	0.06515 ± 0.00065
C1	0.07922 ± 0.00150 ^A^	0.07383 ± 0.00042	0.06900 ± 0.00074	0.06503 ± 0.00090	0.06306 ± 0.00063

Abbreviations: C, ceria; Ra, surface roughness.

Note: The same superscript letters represent no significant difference between group ( *p* > 0.05) by two-way repeated analysis of variance and Bonferroni correction.

**Fig. 2:**
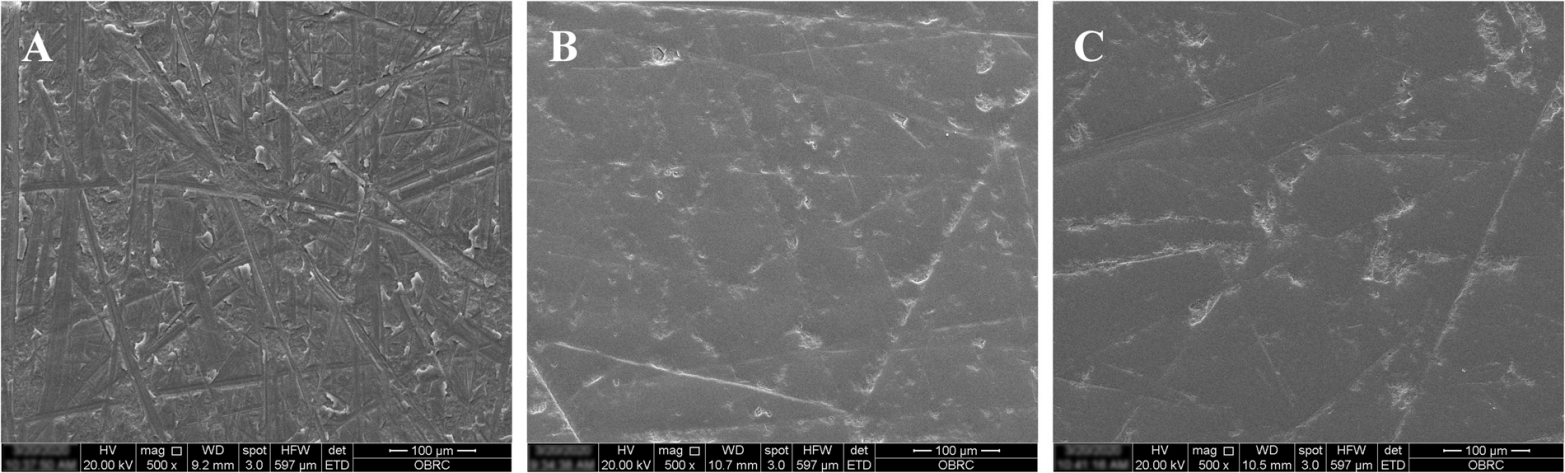
Scanning electron microscope images (×500) of the sample surfaces polished with the different polishing pastes, ( **A** ) surface before polishing, ( **B** ) C0.5 and (C) C1. C, Ceria.

The Ra of the samples observed by scanning electron microscopy (SEM; Fig. 2 ) correlated with their Ra values. The surface of the samples at baseline was the roughest. After polishing, the C1 group had a smoother surface compared with the C0.5 group.

The adsorption experiment results revealed that silicon was not found in the blank test (deionized water); however, it was found in the solution after soaking the specimens in deionized water (0.1037 ± 0.0019 mg/L). After adding ceria particles, the silicon concentration in the filtered solution decreased to 0.0036 ± 0.0013 mg/L ( Table 2 ). There was a significant difference ( *p* < 0.05) in mean silicon concentration between before and after adding the ceria particles.

**Table 2:** The mean silicon concentrations before and after adding cerium oxide particles

Solution	Silicon concentration (mg/L)	
	Before	After added cerium oxide
Blank test	0.00	0.00
Lithium disilicate	0.1037 ± 0.0019	0.0036 ± 0.0013

## Discussion

In the polishing experiment, we evaluated whether a CeO _2_ polishing paste could polish lithium disilicate glass ceramic. The Ra of the C0.5 and C1 groups were significantly lower ( *p <* 0.05) than the baseline roughness. The Ra was significantly decreased ( *p <* 0.05) when the ceria ratio was increased from 0.5 to 1. The surface morphology observed in the SEM analysis correlated with the Ra values. The baseline roughness that had the highest Ra value, demonstrated the roughest surface. The C1 group that had the lowest Ra value demonstrated the smoothest surface. Based on these results, the first null hypothesis was rejected. The results indicated that lithium disilicate ceramic can be polished by the ceria polishing pastes evaluated in this study. Suratwala et al reported that increasing the slurry ceria concentration resulted in decreased atomic force microscope roughness. 16 Moreover, Wang et al reported that increasing the ratio of ceria particles resulted in an increased polishing rate. At low concentration, the chemical formation rate was slow and limited the overall polishing rate. Increased ceria particles led to an increased number of active particles and chemical reactions which is involved in the polishing rate. 17

A previous study reported that water is an important factor in glass polishing due to the presence of hydroxyl groups. The polishing rate increased with increased hydroxyl reactivity. The rate was nearly zero in hydrocarbon liquids. The highest polishing rate was found when water was present. 10 Therefore, deionized water was used as a lubricant in this study. Moreover, Plakhova et al reported that CeO _2_ reacted with water to form surface functional groups on its particules which were important in the CMP process. The reaction between ceria and water is described in **Eq. (1)** . 18

CeO _2_ + 2H _2_ O → Ce(OH) _4_ (1)

Lithium disilicate is a particle-filled glass ceramic. Its structure is approximately 70 vol% lithium disilicate crystals embedded in a glass matrix. Lithium disilicate is composed of 57.0 to 80.0% SiO _2_ , 9.0 to 11.0% Li _2_ O, 0.0 to 13.0% K _2_ O, 0.0 to 11.0% P _2_ O _5_ , 0.0 to 8.0% ZrO _2_ , 0.0 to 8.0% ZnO, 0.0 to 5.0% Al _2_ O _3_ , 0.0 to 5.0% MgO, and 0.0 to 8.0% coloring oxides 13 14 The adsorption experiment results demonstrated that silica was found in the solutions that the lithium disilicate specimen was soaked in. These results indicate that there was a chemical reaction between lithium disilicate and water resulting in its dissolution. Corresponding to previous studies, dental ceramic was found to dissolve in aqueous solutions. 19 20 The reaction between silica (in lithium disilicate ceramic) and water is described in **Eq. (2)** . In water, silica forms a surface functional group which is silanol or ≡Si–OH. The rate of surface removal was controlled by the hydrolysis of the siloxane network and the rate of the reaction below the surface is controlled by the diffusion of water in silica.

SiO _2_ + 2H _2_ O ⇄ Si(OH) _4_

or ≡Si–O–Si≡ + H _2_ O ⇄ 2(≡Si–OH) (2)

The adsorption assay results revealed that the mean silicon concentration of the solution after adding ceria particles and filtering was significantly lower ( *p* < 0.05) than the soaked lithium disilicate specimen solution. Based on these results, the second null hypothesis was rejected. The decreased silicon concentration in the solution after adding ceria particles indicated that silicon was adsorbed by ceria particles.

The adsorption of silicon onto the ceria surfaces was confirmed in our pilot study. The silicon removed from the filtered solution was found in the filtered ceria particles that were analyzed using X-ray fluorescence. However, there was no silicon in the control solution (ceria particles soaked in deionized water). These findings indicate that the silicon was adsorbed by ceria particles.

According to **Eqs. (1)** and **(2)** , the surface functional groups of the ceria particle and silica in water are cerium hydroxide (≡Ce–OH) and silanol (≡Si–OH), respectively. During polishing, ceria particles are in contact with the glass surface. A ≡Si–O–Ce≡ bridging bond forms at the interface as a complex. The reaction is described in **Eq. (3)** .

≡Si–OH + ≡Ce–OH → ≡Si–O–Ce≡ + H _2_ O

or ≡Si–O–Si–OH + ≡Ce–O–Ce–OH → ≡Si–O–Si–O–Ce–O–Ce≡ + H _2_ O (3)

The bond strength of the ≡Si–O–Ce≡ complex is stronger than that of ≡Si–O–Si≡ (in silica). When the ceria particles receive the mechanical polishing force, strain is placed on the ≡Si–O–Ce≡ complex. If the strain is high enough, the bond between ≡Si–O–Si≡ (silica) and ≡Si–O–Ce≡ complex will break. The ≡Si–O–Ce≡ is removed from the glass surface and then the new unreacted surface is exposed. The CMP process cycle then repeats. 10 12 21 22 23 This reaction is described in **Eq. (4)** .

≡Si–O–Si–O–Ce–O–Ce≡ + H _2_ O → ≡Si–OH + HO–Si–O–Ce–O–Ce≡ (4)

The present study investigated the CMP ability of CeO _2_ as an abrasive to polish lithium disilicate glass ceramic. The mechanical factors that affect material removal are abrasive type, size and shape, load during polishing, and polishing speed. 12 24 25 26 27 28 However, CeO _2_ powder with particles <5-µm diameter was the only abrasive type used in this study. Load during polishing and polishing speed were fixed as controlled variables. Moreover, the pH of the polishing slurry, point-of-zero charge, and temperature also affect material removal. 21 22 29 To improve the efficiency of the polishing paste, these factors require further investigation.

In the last decade, the use of CeO _2_ has tremendously increased in many fields, including as a fuel additive, electronics, medicine, ceramic application, polishing agent, and agriculture. 9 However, some reports demonstrated that CeO _2_ escapes into the environment from sludge leakage and wastewater discharge and enter the food chain. 30 31 Donovan et al also reported that CeO _2_ particles were found in drinking water. 32 Therefore, humans may unintentionally eat food or drink contaminated with CeO _2_ . The present study has demonstrated that CeO _2_ particles react with silica in ceramic materials resulting in material removal. A long-term study is needed to investigate the effect of food or drink containing CeO _2_ on dental ceramic restorations in vivo.

## Limitation

The limitation of this study is that it was performed in vitro. There is no report of the toxicity of CeO _2_ in humans. 33 34 However, the safety of using CeO _2_ paste needs to be confirmed before using it clinically as an intraoral polishing paste. The efficiency of a polishing paste might be different under clinical conditions. Moreover, different types of material and polishing protocols may affect the polishing results. Further study is needed to evaluate the mechanical action and chemical reaction of other ceramic materials and other polishing protocols.

## Conclusion

Polishing lithium disilicate with ceria polishing paste generated a significantly lower Ra value and a smoother surface compared with baseline roughness because ceria particles have a mechanical action and chemical react with silica on the lithium disilicate surface. Therefore, it can be used as a CMP paste to create a smooth surface.
